# Tobacco smoking and tuberculosis treatment outcomes: a prospective cohort study in Georgia

**DOI:** 10.2471/BLT.14.147439

**Published:** 2015-03-30

**Authors:** Medea Gegia, Matthew J Magee, Russell R Kempker, Iagor Kalandadze, Tsira Chakhaia, Jonathan E Golub, Henry M Blumberg

**Affiliations:** a University Research Company LLC Branch in Georgia, *United States Agency for International Development*Georgia Tuberculosis Prevention Project, Tbilisi, Georgia.; b Division of Epidemiology and Biostatistics, School of Public Health, Georgia State University, One Park Place NE, Atlanta, GA 30303, United States of America (USA).; c Department of Medicine, Emory University School of Medicine, Atlanta, USA.; d Gudushauri National Medical Centre, Tbilisi, Georgia.; e School of Medicine, Johns Hopkins University, Baltimore, USA.

## Abstract

**Objective:**

To assess the effect of tobacco smoking on the outcome of tuberculosis treatment in Tbilisi, Georgia.

**Methods:**

We conducted a prospective cohort study of adults with laboratory-confirmed tuberculosis from May 2011 to November 2013. History of tobacco smoking was collected using a standardized questionnaire adapted from the global adult tobacco survey. We considered tuberculosis therapy to have a poor outcome if participants defaulted, failed treatment or died. We used multivariable regressions to estimate the risk of a poor treatment outcome.

**Findings:**

Of the 591 tuberculosis patients enrolled, 188 (31.8%) were past smokers and 271 (45.9%) were current smokers. Ninety (33.2%) of the current smokers and 24 (18.2%) of the participants who had never smoked had previously been treated for tuberculosis (*P* < 0.01). Treatment outcome data were available for 524 of the participants, of whom 128 (24.4%) – including 80 (32.9%) of the 243 current smokers and 21 (17.2%) of the 122 individuals who had never smoked – had a poor treatment outcome. Compared with those who had never smoked, current smokers had an increased risk of poor treatment outcome (adjusted relative risk, aRR: 1.70; 95% confidence interval, CI: 1.00–2.90). Those who had ceased smoking more than two months before enrolment did not have such an increased risk (aRR: 1.01; 95% CI: 0.51–1.99).

**Conclusion:**

There is a high prevalence of smoking among patients with tuberculosis in Georgia and smoking increases the risk of a poor treatment outcome.

## Résumé

**Objectif:**

Évaluer l'effet du tabagisme sur les résultats des traitements antituberculeux à Tbilissi, en Géorgie.

**Méthodes:**

Nous avons réalisé une étude de cohorte prospective chez des adultes atteints de tuberculose confirmée en laboratoire, de mai 2011 à novembre 2013. Les antécédents de tabagisme ont été recueillis à l'aide d'un questionnaire standardisé, adapté de l'enquête mondiale sur le tabagisme des adultes. Nous avons considéré que les résultats du traitement antituberculeux étaient mauvais si les participants ne l'avaient pas respecté, n'avaient pas répondu au traitement ou étaient décédés. Nous avons utilisé des régressions multivariées pour estimer le risque de mauvais résultats thérapeutiques.

**Résultats:**

Sur les 591 patients atteints de tuberculose, 188 (31,8%) étaient d'anciens fumeurs et 271 (45,9%) fumaient encore. Quatre-vingt-dix (33,2%) fumeurs actuels et 24 (18,2%) participants n'ayant jamais fumé avaient précédemment pris un traitement contre la tuberculose (P < 0,01). Les données sur les résultats des traitements étaient disponibles pour 524 des participants, sur lesquels 128 (24,4%) – dont 80 (32,9%) des 243 fumeurs actuels et 21 (17,2%) des 122 personnes n'ayant jamais fumé – montraient de mauvais résultats thérapeutiques. Comparés aux personnes n'ayant jamais fumé, les fumeurs actuels présentaient un risque accru de mauvais résultats thérapeutiques (risque relatif ajusté, RRa : 1,70 ; intervalle de confiance de 95%, IC : 1,00-2,90). Les personnes qui avaient arrêté de fumer plus de deux mois avant leur inclusion à l'étude ne présentaient pas une telle augmentation du risque (RRa : 1,01 ; IC de 95% : 0,51-1,99).

**Conclusion:**

Il existe une forte prévalence de tabagisme chez les patients atteints de tuberculose en Géorgie et le tabagisme augmente le risque de mauvais résultats thérapeutiques.

## Resumen

**Objetivo:**

Evaluar los efectos del consumo de tabaco en los resultados del tratamiento de la tuberculosis en Tbilisi, Georgia.

**Métodos:**

Se llevó a cabo un estudio de cohortes prospectivo en adultos con tuberculosis confirmada mediante pruebas de laboratorio de mayo de 2011 a noviembre de 2013. Se recopiló información sobre el consumo de tabaco mediante un cuestionario normalizado adaptado a partir de la Encuesta Mundial sobre el Tabaco y los Adultos. Se consideró que los resultados de la terapia antituberculosa eran deficientes si los participantes incumplían el tratamiento, no lo realizaban o morían. Se utilizaron regresiones multivariables para estimar el riesgo de obtener unos resultados deficientes del tratamiento.

**Resultados:**

De los 591 pacientes con tuberculosis inscritos, 188 (31,8%) eran antiguos fumadores y 271 (45,9%) eran fumadores actuales. Noventa (33,2%) de los fumadores actuales y 24 (18,2%) de los participantes que no habían fumado nunca habían recibido tratamiento para la tuberculosis anteriormente (P < 0,01). Los datos sobre los resultados del tratamiento estuvieron disponibles para 524 de los participantes, de los cuales 128 (24,4%) (incluyendo los 80 (32,9%) de los 243 fumadores y 21 (17,2%) de las 122 personas que nunca habían fumado) obtuvieron unos resultados deficientes del tratamiento. En comparación con las personas que no habían fumado nunca, los fumadores actuales tenían un riesgo superior de obtener unos resultados deficientes del tratamiento (riesgo relativo ajustado, RRa: 1,70; intervalo de confianza, IC, del 95%: 1,00–2,90). Aquellas personas que habían dejado de fumar más de dos meses antes de la inscripción no experimentaban un aumento del riesgo tan elevado (RRa: 1,01 (IC del 95%: 0,51–1,99).

**Conclusión:**

Existe una elevada prevalencia de consumo de tabaco entre los pacientes con tuberculosis en Georgia y el consumo de tabaco aumenta el riesgo de obtener unos resultados deficientes del tratamiento.

## ملخص

**الغرض:**

لتقييم التأثير الناتج عن تدخين التبغ على حصيلة علاج مرض السل في تبلّيسي، جورجيا.

**الطريقة:**

لقد أجرينا دراسة أترابية استباقية على البالغين الذين يعانون من مرض السل المؤكد مختبريًا في الفترة ما بين أيار/مايو 2011 إلى تشرين الثاني/نوفمبر 2013. وقد تم تجميع تاريخ تدخين التبغ باستخدام استبيان معياري معتمد من المسح العالمي لاستهلاك التبغ لدى البالغين. لقد وجدنا أن علاج السل ينتج عنه حصيلة سيئة إذا تخلف المشاركون عن المعالجة، أو فشل علاجهم، أو توفوا. لقد استخدمنا تحوفات متعددة المتغيرات لتقدير الاختطار الناتج عن حصيلة العلاج السيئة.

**النتائج:**

من بين 591 من مرضى السل المسجلين، كان 188 (31.8٪) من المرضى مدخنين سابقين وكان 271 (45.9٪) من المرضى مدخنين حاليين. لقد سبق لتسعين (33.2٪) من المدخنين الحاليين، و 24 (18.2٪) من المشاركين الذين لم يدخنوا أبدًا، العلاج من مرض السل (الاحتمال < 0.01). وتوفرت بيانات حصيلة العلاج لعدد 524 من المشاركين، من بينهم 128 (24.4٪) كانت حصيلة العلاج الخاصة بهم سيئة، بما في ذلك 80 (32.9٪) من 243 من المدخنين الحاليين و21 (17.2٪) من 122 من الأفراد الذين لم يدخنوا أبدًا. مقارنة مع أولئك الذين لم يدخنوا أبدًا، كان هناك اختطار زائد على المدخنين الحاليين لحدوث حصيلة علاج سيئة (الاختطار النسبي المصحح، aRR: 1.70؛ بنسبة أرجحية مقدارها 95%: 1.00 إلى 2.90).أولئك الذين قد أقلعوا عن التدخين لمدة أكثر من شهرين قبل التسجيل، لم يكن لديهم مثل ذلك الاختطار الزائد (:aRR 1.01؛ بنسبة أرجحية مقدارها 95%: 0.50 إلى 1.99).

**الاستنتاج:**

هناك انتشار مرتفع للتدخين بين المرضى الذين يعانون من مرض السل في جورجيا، ويزيد التدخين من اختطار حصيلة العلاج السيئة.

## 摘要

**目的:**

旨在评价格鲁吉亚第比利斯境内吸烟对结核病治疗效果的影响。

**方法:**

从 2011 年 5 月至 2013 年 11 月，我们在经实验室确诊患有结核病的成人中开展了一项前瞻性群组研究，并且通过改编自全球成人烟草调查的标准化问卷收集了吸烟史资料。我们认为如果参与者违约、不进行治疗或死亡，那么结核病治疗会有不良的效果。我们采用多变量回归分析的方法评估了不良治疗效果的风险。

**结果:**

在参与调查的 591 名结核病患者中，其中 188 名 (31.8%) 是既往吸烟者，271 名 (45.9%) 是目前吸烟者。目前吸烟者中有 90 名 (33.2%)、从未吸烟者中有 24 名 (18.2%) 曾在之前因结核病接受过治疗 (P < 0.01)。治疗效果数据对 524 名参与者均为有效可用，其中 128 名 (24.4%) 参与者的治疗效果不良，包括 243 名目前吸烟者中的 80 名 (32.9%) 和 122 名从未吸烟者中的 21 名 (17.2%)。与从未吸烟者相比，目前吸烟者产生不良治疗效果的风险较高（调整过的相对风险，aRR：1.70；95% 置信区间，CI：1.00–2.90）。那些在参与调查之前已戒烟两个月以上的人并未增加该风险 (aRR:1.01；95% CI： 0.51–1.99)。

**结论:**

格鲁吉亚境内结核病患者吸烟流行率很高，并且吸烟增加了产生不良治疗效果的风险。

## Резюме

**Цель:**

Оценка воздействия табакокурения на исход лечения туберкулеза в Тбилиси, Грузия.

**Методы:**

В период с мая 2011 г. по ноябрь 2013 г. было проведено проспективное когортное исследование взрослого населения с диагнозом туберкулеза, подтвержденным данными лабораторных анализов. С помощью стандартизированной анкеты, разработанной на основе Глобального опроса взрослого населения о потреблении табака (GATS), была собрана история табакокурения участников. Исход лечения туберкулеза рассматривался нами как плохой, если участник исследования не доводил лечение до конца, лечение не было эффективным или участник исследования умирал. Для оценки риска плохого исхода лечения использовались многопараметрические регрессии.

**Результаты:**

В исследовании принял участие 591 пациент с туберкулезом, из которых 188 человек (31,8 %) ранее были курильщиками, а 271 человек (45,9 %) курил на момент начала исследования. Девяносто человек (33,2 %) из числа активных курильщиков и 24 человека (18,2 %) из числа тех участников, которые никогда не курили, ранее получали лечение от туберкулеза (P < 0,01). Данные по исходу лечения были доступны для 524 участников, из которых исход лечения был плохим у 128 человек (24,4 %). В это число входили 80 человек (32,9 %) из числа 243 активных курильщиков и 21 (17,2 %) из тех 122, кто никогда не курил. По сравнению с теми, кто никогда не курил, у активных курильщиков был выявлен повышенный риск плохого исхода лечения (скорректированный относительный риск, ОР: 1,70; 95 % доверительный интервал, ДИ: 1,00–2,90). У тех, кто бросил курить более чем за два месяца до вступления в исследование, риск был не таким высоким (скорректированный ОР: 1,01; 95 % ДИ: 0,51–1,99).

**Вывод:**

У пациентов с туберкулезом в Грузии широко распространено курение, и курение увеличивает риск плохого исхода лечения.

## Introduction

Both tobacco smoking and tuberculosis are major global public health problems. Globally, nearly 6 million people died from tobacco use in 2011 and tobacco use is estimated to be responsible for 16% of deaths among men and 7% of deaths among women each year.^1^^,^^2^ In 2012, there were an estimated 8.6 million new tuberculosis cases and 1.3 million tuberculosis-related deaths worldwide.^2^ Smoking is common in the 22 countries categorized by the World Health Organization (WHO) as high-burden countries for tuberculosis – which together account for more than 80% of all tuberculosis cases. The burden of smoking among patients with tuberculosis is poorly defined in most countries.^2^

An understanding of the epidemiological relationship between smoking and tuberculosis is important because both smoking and tuberculosis cause extensive morbidity and mortality worldwide. Compared with those who have never smoked, it is estimated that people who smoke have approximately twice the risk of both *Mycobacterium tuberculosis* infection^3^ and active tuberculosis.^4^ However, data on the impact of smoking on treatment outcomes among patients with active tuberculosis are limited.^5^

Georgia has a high incidence of tuberculosis, a high incidence of multidrug-resistant (MDR) tuberculosis and a high prevalence of smoking.^6^^,^^7^ In 2012, for example, there were 116 cases of tuberculosis per 100 000 people and MDR tuberculosis accounted for 9.2% of the new cases and 31.0% of the retreatment cases.^6^ In 2010, a national survey indicated that about 52.8% of Georgian men – including 64.0% of those aged 30–49 years – and 6.1% of Georgian women were smokers.^7^ The main objectives of this study were to estimate the prevalence of smoking and the impact of smoking on tuberculosis treatment outcomes among patients with tuberculosis in the Georgian capital of Tbilisi.

## Methods

### Design and study population

A prospective cohort study was conducted between May 2011 and November 2013 among people attending the National Centre for Tuberculosis and Lung Diseases, or one of its affiliated outpatient clinics, in Tbilisi, Georgia. To be eligible for enrolment, a patient had to be aged at least 18 years, have provided a sputum specimen that had been found either smear-positive for acid-fast bacilli or culture-positive for *Mycobacterium tuberculosis* and have started directly-observed, standard tuberculosis therapy according to WHO guidelines^8^ within the previous two months. Eligible patients were enrolled between May 2011 and February 2012. In November 2013, details of the participants’ treatment outcomes were collected from the database of patient records maintained by the National Centre for Tuberculosis and Lung Diseases.

At the time of study enrolment, after the patients had provided written informed consent, our research team conducted face-to-face interviews of eligible inpatients and outpatients. Although most of the data we used were collected using a standardized questionnaire adapted from the one employed in the global adult tobacco survey,^9^ additional covariates of interest were collected from the National Centre for Tuberculosis and Lung Diseases database. Self-reported information on age, alcohol use, socioeconomic indicators, prison history, human immunodeficiency virus (HIV) status, exposure to a person with MDR tuberculosis and tuberculosis symptoms – i.e. cough with and without sputum – were recorded on the questionnaire. Previous tuberculosis treatment, results from the examination of a baseline sputum smear and current tuberculosis treatment regimen were obtained from the National Centre for Tuberculosis and Lung Diseases database.

### Study definitions

History of smoking was used as the primary exposure variable. Patients were first asked if they currently smoked on a daily basis, less than daily, or not at all. Those who reported that they currently smoked – either daily or less than daily – were categorized as current smokers in the primary analyses. The remaining patients were then asked if they had smoked in the past on a daily or less than daily basis and those giving a positive response were categorized as past smokers in the primary analyses. Patients who said that they had never smoked were always categorized as never smokers.

In our secondary analyses, we used other measures of tobacco use. Patients who currently smoked on a daily basis and patients who currently smoked on a less than daily basis were separately compared with past and never smokers. Patients also reported smokeless tobacco use and frequency of household second-hand exposure to tobacco smoke. To account for possible misclassification of those patients who had stopped smoking once they developed tuberculosis symptoms,^10^^,^^11^ we also used modified definitions for smoking status. For example, we put patients who had ceased smoking in the two or four months before enrolment in separate categories and reclassified all such patients as current smokers instead of past smokers.^12^^,^^13^

The outcome of each participant’s most recent treatment for tuberculosis was used as the primary study outcome. Treatment outcomes were categorized according to WHO definitions.^6^ A patient who was cured or completed treatment was defined as having a favourable outcome whereas a patient who defaulted, failed treatment or died was defined as having a poor outcome.

Laboratory studies were performed at the Georgia National Tuberculosis Reference Laboratory. Since 2005, the quality of the services provided by this laboratory has been assessed annually by the WHO’s Supranational Tuberculosis Reference Laboratory in Antwerp, Belgium.^14^ A standard semiquantitative scale was used to classify the number of acid-fast bacilli present in sputum smears.^15^ Sputum samples were tested for *M. tuberculosis* by culture on Lowenstein–Jensen solid medium and using BACTEC MGIT 960 mycobacterial detection system (BD, Franklin Lakes, United States of America).^14^ Isolated samples of *M. tuberculosis* were tested for their sensitivity to first-line anti-tuberculosis drugs using either the absolute concentration method or the BACTEC MGIT 960 mycobacterial detection system but their sensitivity to second-line drugs was investigated using proportion methods.^14^

### Data analyses

Study data were entered into a REDCap^16^ electronic database and analysed using SAS 9.3 (SAS Institute, Cary, USA). Bivariate associations were analysed using *χ^2^* tests for categorical variables and the Wilcoxon rank sum or Kruskal–Wallis tests for continuous variables. A two-sided *P*-value of less than 0.05 was considered statistically significant. Binomial regression models were used to estimate risk ratios (RR) and 95% confidence intervals (CI) for a poor tuberculosis treatment outcome. Potential confounders – based on significant bivariate associations with the primary exposure and outcome, previous literature or directed acyclic graph theory^17^ – were included in the regression models. We used the primary multivariable model to estimate the effect of current smoking status on risk of poor tuberculosis outcome. For our secondary models we used alternative definitions of current smoking status but included the same covariates as in the primary model. To assess the distribution of missing outcome data, we compared the sociodemographic, tuberculosis and smoking characteristics of the patients with known treatment outcomes with those of the other patients. The study was reviewed and approved by the institutional review boards of the Georgia National Centre for Tuberculosis and Lung Diseases (Tbilisi, Georgia), Emory University (Atlanta, USA) and the Johns Hopkins Bloomberg School of Public Health (Baltimore, USA).

## Results

Overall, 591 (64.4%) of 917 eligible patients with tuberculosis were invited to participate in our study and all agreed to be enrolled. Of the participants, 457 (77.3%) were male. The median age was 36 years (interquartile range, IQR: 26–50 years), their median monthly income was 118 United States dollars (US$) (IQR: US$ 59–294) and 465 (78.7%) of them had been educated to at least high school level. In November 2013, information on tuberculosis treatment outcome was available for 524 (88.7%) of the participants.

### Smoking characteristics

Of the participants, 271 (45.9%) were current smokers and 188 (31.8%) were past smokers (Table 1). Most of the current and past smokers were male. Current smokers were older, with fewer years of education and lower income compared to never smokers (Table 1). Previous tuberculosis treatment, cough with sputum and exposure to a case of MDR tuberculosis were more frequently reported by current smokers than never smokers (Table 1). Compared with the never smokers, past smokers were significantly less likely to have MDR tuberculosis (Table 1).

**Table 1:** Baseline characteristics of adults with active tuberculosis, Georgia, 2011–2012

Characteristic	No. (%)^a^			*P*
	Never smokers (*n* = 132)	Past smokers (*n* = 188)	Current smokers (*n* = 271)	
**Sex**				
Female	89 (67.4)	26 (13.8)	19 (7.0)	< 0.01
Male	43 (32.6)	162 (86.2)	252 (93.0)	
**Age (years)**				
18–24	47 (35.6)	39 (20.7)	30 (11.1)	< 0.01
25–34	38 (28.8)	42 (22.3)	73 (26.9)	
35–54	24 (18.2)	80 (42.6)	128 (47.2)	
≥ 55	23 (17.4)	27 (14.4)	40 (14.8)	
**Education (years)**				
≤ 9	29 (22.0)	33 (17.5)	64 (23.6)	< 0.01
10	33 (25.0)	77 (41.0)	118 (43.5)	
≥ 11	70 (53.0)	78 (41.5)	89 (32.8)	
**Monthly income (US$)**				
≤ 65	25 (18.9)	59 (31.4)	95 (35.1)	0.02
66–200	49 (37.1)	50 (26.6)	86 (31.7)	
≥ 201	48 (36.4)	59 (31.4)	72 (26.6)	
Unknown	10 (7.6)	20 (10.6)	18 (6.6)	
**Internally displaced**				
No	123 (93.2)	173 (92.0)	250 (92.3)	0.92
Yes	9 (6.8)	15 (8.0)	21 (7.7)	
**Prison history**				
No	127 (96.2)	163 (86.7)	181 (66.8)	< 0.01
Yes	5 (3.8)	25 (13.3)	90 (33.2)	
**Frequency of alcohol use (days/week)**				
0	74 (56.1)	50 (26.6)	51 (18.8)	< 0.01
< 1	45 (34.1)	61 (32.5)	75 (27.7)	
1–2	9 (6.8)	42 (22.3)	66 (24.4)	
≥ 3	2 (1.5)	26 (13.8)	76 (28.0)	
Unknown	2 (1.5)	9 (4.8)	3 (1.1)	
**Self-reported HIV status**				
Negative	117 (88.6)	168 (89.4)	243 (89.7)	0.86
Positive	2 (1.5)	3 (1.6)	3 (1.1)	
Unknown	13 (9.9)	17 (9.0)	25 (9.2)	
**Smokeless tobacco use**				
No	95 (72.0)	147 (78.2)	195 (72.0)	0.39
Yes	1 (0.7)	4 (2.1)	5 (1.8)	
Unknown	36 (27.3)	37 (19.7)	71 (26.2)	
**Second-hand exposure to smoke in household**				
Never	49 (37.1)	97 (51.6)	108 (39.9)	< 0.01
Less than daily	20 (15.1)	17 (9.0)	22 (8.1)	
Daily	62 (47.0)	68 (36.2)	118 (43.5)	
Unknown	1 (0.8)	6 (3.2)	23 (8.5)	
**Previous tuberculosis treatment**				
No	108 (81.8)	147 (78.2)	181 (66.8)	< 0.01
Yes	24 (18.2)	41 (21.8)	90 (33.2)	
**Result of baseline sputum examination**				
Negative for AFB	65 (49.2)	85 (45.2)	102 (37.6)	0.06
Positive for AFB	67 (50.8)	103 (54.8)	169 (62.4)	
**Self-reported cough**				
No	31 (23.5)	45 (23.9)	53 (19.6)	0.03
Yes	101 (76.5)	139 (73.9)	218 (80.4)	
Unknown	0 (0.0)	4 (2.1)	0 (0.0)	
**Self-reported cough with sputum**				
No	28 (21.2)	12 (6.4)	21 (7.7)	< 0.01
Yes	71 (53.8)	121 (64.4)	191 (70.5)	
Unknown	33 (25.0)	55 (29.3)	59 (21.8)	
**Self-reported exposure to case of MDR tuberculosis**				
No	97 (73.5)	139 (73.9)	158 (58.3)	< 0.01
Yes	35 (26.5)	44 (23.4)	111 (41.0)	
Unknown	0 (0.0)	5 (2.7)	2 (0.7)	
**Type of tuberculosis**				
Drug-susceptible	78 (59.1)	131 (69.7)	170 (62.7)	< 0.01
MDR	52 (39.4)	46 (24.5)	95 (35.1)	
Unknown	2 (1.5)	11 (5.9)	6 (2.2)	
**Median values**				
Age, years (IQR)	29.0 (22.0–43.0)	36.0 (25.0–51.0)	39.0 (29.0–50.0)	< 0.01
Education, years (IQR)	11.0 (10.0–11.0)	10.0 (10.0–11.0)	10.0 (10.0–11.0)	< 0.01
Monthly income, US$ (IQR)	176.5 (88.2–294.1)	117.6 (58.8–294.1)	117.6 (41.2–235.3)	< 0.01

AFB: acid-fast bacilli; HIV: human immunodeficiency virus; IQR: interquartile range; MDR: multidrug-resistant; US$: United States dollars.

^a^ Unless indicated otherwise.

### Treatment outcomes

We were unable to determine the final treatment outcomes of 67 participants because of missing data (34) or because, at the end of our study, the participants remained on second-line therapy against MDR tuberculosis (33). We collected information on the final treatment outcomes of the remaining 524 participants. Of these participants, 128 (24.4%) had a poor outcome – 99 defaulted, 11 failed treatment and 18 died (Table 2). In terms of their demographic characteristics, baseline smear status and smoking characteristics, the 524 participants with known treatment outcomes did not appear to be significantly different to the  67 other participants.

**Table 2:** Smoking status and treatment outcomes among adults with tuberculosis, Georgia, 2011–2012

Treatment outcome^a^	No. (%)		
	Never smokers (*n* = 122)	Past smokers (*n* = 159)	Current smokers (*n* = 243)
**Favourable**	101 (82.8)	132 (83.0)	163 (67.1)
Cured	42 (34.4)	69 (43.4)	81 (33.3)
Completed treatment	59 (48.4)	63 (39.6)	82 (33.7)
**Poor**	21 (17.2)	27 (17.0)	80 (32.9)
Defaulted treatment	16 (13.1)	22 (13.8)	61 (25.1)
Failed treatment	1 (0.8)	3 (1.9)	7 (2.9)
Died	4 (3.3)	2 (1.3)	12 (4.9)

^a^ As defined by the World Health Organization^6^ and recorded six months after the initiation of treatment.

Note: Inconsistencies arise in some values due to rounding.

In our unadjusted analyses, the risk of poor treatment outcomes was higher among current smokers (RR: 1.91; 95% CI: 1.25–2.94), males (RR: 1.74; 95% CI: 1.12–2.71), those with a history of imprisonment (RR: 1.46; 95% CI: 1.05–2.03), previous tuberculosis treatment (RR: 1.94; 95% CI: 1.45–2.61), sputum-smear-positive at the baseline check (RR: 2.33; 95% CI: 1.61–3.37) or MDR tuberculosis (RR: 4.53; 95% CI: 3.30–6.21) (Table 3). In the primary multivariable analysis, being a current smoker (RR: 1.70; 95% CI: 1.00–2.90) and receiving treatment for MDR tuberculosis (RR: 3.12; 95% CI: 2.30–4.22) remained significantly associated with a poor outcome (Table 3). We did not detect any one-way statistical interactions between smoking status and any of the other covariates that we included in the adjusted model.

**Table 3:** Association between baseline patient characteristics and risk of poor tuberculosis treatment outcomes, Georgia, 2011–2012

Characteristic	No. of patients with poor/known outcome (%)	Risk ratio (95% CI)^a^	
		Crude	Adjusted^b^
**Sex**			
Female	19/122 (15.6)	1.00	1.00
Male	109/402 (27.1)	1.74 (1.12–2.71)	1.12 (0.67–1.86)
**Age (years)**			
18–24	22/109 (20.2)	1.00	1.00
25–34	32/134 (23.9)	1.18 (0.73–1.91)	1.04 (0.68–1.58)
35–54	49/199 (24.6)	1.22 (0.78–1.90)	0.86 (0.60–1.25)
≥ 55	25/82 (30.5)	1.51 (0.92–2.48)	1.17 (0.83–1.64)
**Education (years)**			
≤ 9	32/114 (28.1)	1.00	–
10	50/197 (25.4)	0.90 (0.62–1.32)	–
≥ 11	46/213 (21.6)	0.77 (0.52–1.14)	–
**Monthly income (US$)**			
≤ 65	36/161 (22.4)	1.00	1.00 (0.99–1.00)^c^
66–200	53/165 (32.1)	1.44 (1.00–2.07)	–
≥ 201	29/156 (18.6)	0.83 (0.54–1.29)	–
Unknown	10/42 (23.8)	–	–
**Internally displaced**			
No	120/488 (24.6)	1.00	–
Yes	8/36 (22.2)	0.90 (0.48–1.70)	–
**Prison history**			–
No	94/420 (22.4)	1.00	1.00
Yes	34/104 (32.7)	1.46 (1.05–2.03)	1.12 (0.82–1.53)
**Frequency of alcohol use (days/week)**			
0	29/158 (18.4)	1.00	1.00
< 1	49/153 (32.0)	1.74 (1.17–2.61)	1.02 (0.72–1.44)^d^
1–2	28/106 (26.4)	1.44 (0.91–2.27)	–
≥ 3	20/94 (21.3)	1.16 (0.70–1.93)	–
Unknown	2/13 (15.4)	–	–
**Previous tuberculosis treatment**			
No	78/394 (19.8)	1.00	1.00
Yes	50/130 (38.5)	1.94 (1.45–2.61)	1.22 (0.94–1.58)
**Result of baseline sputum examination**			
Negative for AFB	30/218 (13.8)	1.00	1.00
Positive for AFB	98/306 (32.0)	2.33 (1.61–3.37)	1.37 (0.99–1.90)
**Self-reported cough**			
No	25/120 (20.8)	1.00	–
Yes	103/401 (25.7)	1.23 (0.84–1.81)	–
Unknown	0/3 (0.0)	–	–
**Self-reported cough with sputum**			
No	11/54 (20.4)	1.00	–
Yes	84/334 (25.1)	1.23 (0.71–2.16)	–
Unknown	33/136 (24.3)	–	–
**Self-reported exposure to case of MDR tuberculosis**			
No	83/350 (23.7)	1.00	–
Yes	44/167 (26.3)	1.11 (0.81–1.52)	–
Unknown	1/7 (14.3)	–	–
**Type of tuberculosis**			
Drug-susceptible	43/364 (11.8)	1.00	1.00
MDR	85/159 (53.5)	4.53 (3.30–6.21)	3.12 (2.30–4.22)
Unknown	0/1 (0.0)	–	–
**Self-reported HIV status**			
Negative	118/464 (25.4)	1.00	1.00
Positive	1/8 (12.5)	0.49 (0.08–3.10)	–
Unknown	9/52 (17.3)	–	–
**Model 1 – smoking status**			
Never smoker	21/122 (17.2)	1.00	1.00
Past smoker	27/159 (17.0)	0.99 (0.59–1.66)	1.24 (0.71–2.17)
Current smoker	80/243 (32.9)	1.91 (1.25–2.94)	1.70 (1.00–2.90)
**Model 2 – smoking status**			
Never smoker	21/122 (17.2)	1.00	–
Past smoker	9/86 (10.5)	0.61 (0.29–1.26)	–
Ceased smoking in previous two months	18/73 (24.7)	1.43 (0.82–2.50)	–
Current smoker	80/243 (32.9)	1.91 (1.25–2.94)	–
**Model 3 – smoking status**			
Never smoker	21/122 (17.2)	1.00	–
Past smoker	9/86 (10.5)	0.61 (0.29–1.26)	–
Current smoker^e^	98/316 (31.0)	1.80 (1.18–2.75)	–
**Model 4 – smoking status**			
Never smoker	21/122 (17.2)	1.00	–
Past smoker	6/70 (8.6)	0.50 (0.21–1.17)	–
Current smoker^f^	101/332 (30.4)	1.77 (1.16–2.69)	–
**Current smoking frequency**			
Zero	48/280 (17.1)	1.00	–
Less than daily	3/13 (23.1)	1.35 (0.48–3.75)	–
Daily	77/231 (33.3)	1.94 (1.42–2.67)	–
**Smokeless tobacco use**			
No	90/385 (23.4)	1.00	–
Yes	2/9 (22.2)	0.95 (0.28–3.27)	–
Unknown	36/130 (27.7)	–	–
**Second-hand exposure to smoke in household**			
Never	52/224 (23.2)	1.00	–
Less than daily	23/52 (44.2)	1.91 (1.29–2.81)	–
Daily	46/221 (20.8)	0.90 (0.63–1.27)	–
Unknown	7/27 (25.9)	–	

AFB: acid-fast bacilli; CI: confidence interval; HIV: human immunodeficiency virus; MDR: multidrug-resistant; US$: United States dollars.

^a^ For a poor treatment outcome – i.e. death or treatment default or failure – recorded six months after the initiation treatment.

^b^ Each ratio was adjusted for all of the other variables for which adjusted odds ratios are given in the table.

^c^ Per US$ 10 increase in monthly income.

^d^ Value for any alcohol use versus none.

^e^ Including patients who ceased smoking before two months of enrolment.

^f^ Including patients who ceased smoking before four months of enrolment.

Compared with never smokers, patients with tuberculosis who had ceased smoking within the two months before enrolment had an increased – but not significantly increased – adjusted risk of a poor treatment outcome (adjusted relative risk, aRR: 1.44; Table 4). In models 3 and 4, we considered patients who had ceased smoking in the two and four months before enrolment as current smokers, respectively, and this reduced the strength of the apparent association between current smoking and a poor treatment outcome – to give adjusted relative risks of 1.67 and 1.64, respectively (Table 4). Patients who, when enrolled, had ceased smoking for more than two months had a similar risk of a poor treatment outcome to those who had never smoked (RR: 1.01; Table 4). There were no significant associations between poor tuberculosis treatment outcomes and smokeless tobacco use or household second-hand exposure to tobacco smoke.

**Table 4:** Adjusted associations between smoking history and risk of poor tuberculosis treatment outcomes, Georgia, 2011–2012

Smoking history	Adjusted risk ratio (95% CI)^a^
**Model 1 – smoking status**	
Never smoker	1.00
Past smoker	1.24 (0.71–2.17)
Current smoker	1.70 (1.00–2.90)
**Model 2 – smoking status**	
Never smoker	1.00
Past smoker	1.01 (0.51–1.99)
Ceased smoking in previous two months	1.44 (0.80–2.57)
Current smoker	1.69 (1.00–2.86)
**Model 3 – smoking status**	
Never smoker	1.00
Past smoker	1.01 (0.50–2.04)
Current smoker^b^	1.67 (0.99–2.83)
**Model 4 – smoking status**	
Never smoker	1.00
Past smoker	0.85 (0.39–1.88)
Current smoker^c^	1.64 (0.98–2.75)
**Model 5 – smoking frequency**	
Never or past smoker	1.00
Less than daily	1.29 (0.52–3.19)
Daily	1.46 (1.05–2.04)
**Model 6 – smokeless tobacco use**	
No	1.00
Yes	1.63 (0.52–5.15)
**Model 7 – second-hand exposure to tobacco smoke in household**	
Never	1.00
Less than daily or daily	1.11 (0.86–1.44)
**Model 8 – smoking status**	
Never or past smoker	1.00
Current smoker	1.45 (1.05–2.01)

CI: confidence interval.

^a^ Adjusted risk ratio for a poor treatment outcome – i.e. death or treatment default or failure – six months after the initiation of treatment. Derived from a multivariable logistic regression model that was adjusted for sex, age, income, prison history, alcohol use, previous tuberculosis treatment, baseline smear status and use of second-line treatment.

^b^ Including patients who ceased smoking before two months of enrolment.

^c^ Including patients who ceased smoking before four months of enrolment.

## Discussion

There is a high prevalence of smoking among patients with tuberculosis in Georgia and, among such patients, smoking significantly increases the risk of a poor treatment outcome. After adjusting for other individual characteristics that are potentially related to both smoking and tuberculosis treatment outcome, we found that the risk of a poor tuberculosis treatment outcome was 70% greater in current smokers compared to never smokers. Patients being treated for MDR tuberculosis had a 3-fold greater risk of a poor outcome compared to patients being treated for other forms of tuberculosis. We also found that patients who had recently stopped smoking had a lower risk of a poor tuberculosis outcome than current smokers.

As the government in Georgia prepares for new national policy on tobacco control, the findings of our study are particularly timely and relevant. In Georgia, it is currently illegal to smoke in medical facilities^18^ but it has been difficult to enforce this legislation. Additional policies are needed to eliminate or at least reduce tobacco use in health-care settings in general and tuberculosis clinics in particular. Future policies on tobacco use in the country should promote smoking cessation programmes for patients with tuberculosis.

Globally, more than 20% of people older than 15 years smoke tobacco, according to WHO data from 2014.^19^ However, the prevalence of smoking among people with tuberculosis is often well above 20%. For example, a South African study observed that 56% of patients with active tuberculosis were current smokers.^20^ Similarly, 54.6% of Chinese patients with tuberculosis were smokers.^13^ In our study, 59.9% of the tuberculosis patients were either current smokers or individuals who had ceased smoking no more than two months earlier. Because smoking induces coughing and other symptoms consistent with tuberculosis, there may be longer delays in the diagnosis of tuberculosis among smokers than among non-smokers. For example, another study in Georgia reported a greater likelihood of prolonged delay (more than 23 days) in active tuberculosis diagnosis in smokers compared to never smokers (adjusted odds ratio, aOR: 3.03; 95% CI: 1.24–7.40).^21^

At the time of our study, few data had been published on the relationship between smoking and treatment outcomes among patients with tuberculosis.^22^^,^^23^ Georgian patients whose sputum samples had been found culture-positive for MDR tuberculosis were less likely to become culture-negative after treatment if they currently smoked, (adjusted hazard ratio: 0.82; 95% CI: 0.71–0.95).^24^ Other studies have shown that smoking is associated with increased risk of tuberculosis mortality,^25^ tuberculosis treatment failure,^26^ and relapse after treatment completion.^27^^,^^28^ In a study in Brazil, it was found that – even after adjusting for confounders – smoking was associated with sputum culture positivity after 60 days of first-line tuberculosis treatment.^23^ Among patients with pulmonary tuberculosis in India, smokers were found to have a threefold greater risk of recurrent tuberculosis than non-smokers.^28^ Current smokers with tuberculosis were more likely to default on their treatment than non-smokers in the Hong Kong Special Administrative Region (aOR: 3.00; 95% CI: 1.41–6.39)^29^ and in Nigeria (odds ratio: 1.61; 95% CI: 1.31–1.98)^30^ In Morocco, one study found that smokers were twice as likely to fail tuberculosis treatment as non-smokers^26^ – although the researchers did not control for important confounders such as previous tuberculosis treatment and use of a second-line treatment regimen.

Biological mechanisms related to smoking that impair host defences and increase the risk of *M. tuberculosis* infection probably contribute to the relatively poor results of tuberculosis treatment among smokers. For example, smoking may have an irreversible inhibitory effect on nitric oxide synthase – the enzyme needed by alveolar macrophages to form nitric oxide to inhibit the multiplication of *M. tuberculosis*.^31^^,^^32^ Cigarette smoking can increase the availability of iron in the lower respiratory tract^33^ and iron may bind with nitric oxide to generate toxic radicals^34^ that can interfere with alveolar macrophages.^35^ Smoking also probably reduces the ability of alveolar macrophages to mount an effective pulmonary immune defence by altering the cells’ expression of pro-inflammatory cytokines.^36^

Our study had several limitations. First, our primary exposure variable – self-reported smoking status – was subject to potential misclassification. It is possible that our participants underreported their tobacco use. However, our questionnaires were administered by the research team and not by the patients’ health-care providers. This should have reduced the likelihood that patients underreported their tobacco use.^37^ Moreover, self-reported measures of tobacco use have been found to be reasonably accurate – with less than 1.5% of respondents claiming not to use tobacco when they are current users.^38^ If we misclassified some smokers as never smokers, our estimate of the effect of smoking on tuberculosis outcomes would be an underestimate and our main findings would remain unchanged. Second, as there are probably patient characteristics associated with both smoking and poor tuberculosis treatment outcomes that we failed to include in our multivariable models, such models may have been affected by residual confounding. However, we did adjust for all of the key factors that have been established as important risks for a poor tuberculosis treatment outcome – e.g. a history of previous tuberculosis treatment, baseline sputum-smear score and the use of a second-line treatment regimen. Third, as our data came from patients with tuberculosis attending treatment facilities in Tbilisi, our findings may not be nationally representative. However, the distributions of patient demographics, smear positivity and previous tuberculosis treatment history seen in our study are similar to those reported by WHO for Georgia.^39^

In conclusion, smoking is an independent risk factor for poor tuberculosis treatment outcomes. Smoking cessation programmes need to be targeted at tuberculosis patients – both by clinicians specializing in tuberculosis and by national tuberculosis control initiatives. The effectiveness of such programmes – in reducing smoking among tuberculosis patients and improving tuberculosis treatment outcomes – also needs to be assessed.
